# Achieving high accuracy in meniscus tear detection using advanced deep learning models with a relatively small data set

**DOI:** 10.1002/ksa.12369

**Published:** 2024-07-17

**Authors:** Erdal Güngör, Husam Vehbi, Ahmetcan Cansın, Mehmet Batu Ertan

**Affiliations:** ^1^ Department of Orthopaedics and Traumatology Medipol University Esenler Hospital Istanbul Turkey; ^2^ Department of Radiology Medipol University Esenler Hospital Istanbul Turkey; ^3^ International School of Medicine İstanbul Medipol University Istanbul Turkey; ^4^ Department of Orthopaedics and Traumatology Medicana International Ankara Hospital Ankara Turkey

**Keywords:** deep learning, magnetic resonance imaging, meniscus tear detection, object detection, YOLOv8

## Abstract

**Purpose:**

This study aims to evaluate the effectiveness of advanced deep learning models, specifically YOLOv8 and EfficientNetV2, in detecting meniscal tears on magnetic resonance imaging (MRI) using a relatively small data set.

**Method:**

Our data set consisted of MRI studies from 642 knees—two orthopaedic surgeons labelled and annotated the MR images. The training pipeline included MRI scans of these knees. It was divided into two stages: initially, a deep learning algorithm called YOLO was employed to identify the meniscus location, and subsequently, the EfficientNetV2 deep learning architecture was utilized to detect meniscal tears. A concise report indicating the location and detection of a torn meniscus is provided at the end.

**Result:**

The YOLOv8 model achieved mean average precision at 50% threshold (mAP@50) scores of 0.98 in the sagittal view and 0.985 in the coronal view. Similarly, the EfficientNetV2 model obtained area under the curve scores of 0.97 and 0.98 in the sagittal and coronal views, respectively. These outstanding results demonstrate exceptional performance in meniscus localization and tear detection.

**Conclusion:**

Despite a relatively small data set, state‐of‐the‐art models like YOLOv8 and EfficientNetV2 yielded promising results. This artificial intelligence system enhances meniscal injury diagnosis by generating instant structured reports, facilitating faster image interpretation and reducing physician workload.

**Level of Evidence:**

Level III.

**Abbreviations**
AI: artificial intelligence
AP: average precision
AUC: area under the curve
CNN: convolutional neural network
FN: false negative
FP: false positive
IoU: intersection over union
mAP: mean average precision
MRI: magnetic resonance imaging
ROIs: regions of interest
TP: true positive

## INTRODUCTION

Meniscus tears, often resulting from trauma or degenerative changes, are a common source of knee pain and dysfunction. Magnetic resonance imaging (MRI) is the standard diagnostic tool for identifying these tears and planning treatment [5, 10]. Besides the suitability of treatment prescriptions, significant diagnostic inaccuracies can affect physically active individuals, potentially leading to unnecessary medical procedures or delays in receiving appropriate care. The advent of automated deep learning‐based tools could support and enhance the diagnostic capabilities of general radiologists and orthopaedic surgeons while reducing the time required for comprehensive MRI assessments. Recent years have seen a variety of studies focused on the classification, detection and segmentation of damaged meniscus tissue [3, 18], as well as educational research aimed at training orthopaedic surgeons in understanding and developing these methodologies and models [15]. Recent years have seen various studies focused on the classification, detection and segmentation of damaged meniscus tissue [4, 21], as well as educational research aimed at training orthopaedic surgeons in understanding and developing these methodologies and models [18].

The purpose of this study is to evaluate the effectiveness of advanced deep learning models, specifically YOLOv8 and EfficientNetV2, in detecting meniscal tears on MRI images using a relatively small data set. We hypothesize that advanced deep learning models, specifically YOLOv8 and EfficientNetV2, can achieve superior diagnostic accuracy and localization precision in detecting meniscal tears using MRI images, even with a relatively small data set. This is clinically relevant as it aims to improve diagnostic accuracy, enable precise and timely treatment planning, reduce diagnostic errors and lower physician workload.

## MATERIALS AND METHODS

### Database creation

This study was approved by the Research Ethics Committee of our hospital. We included patients aged between 30 and 45 years who underwent MRI examinations from 2022–2024 at our hospital. Patients with a history of knee surgery were excluded. We collected MRI results from 642 individuals, with 433 diagnosed with a torn meniscus and 209 without. For each individual, we obtained two slices: one sagittal and one coronal. These slices were chosen because they best displayed the meniscus lesion. The specific MRI sequences used were T2‐weighted and proton density (PD)‐weighted sequences, which are optimal for visualizing meniscal tears. For this study, we chose EfficientNetV2 for image classification and YOLOv8 for object detection due to their state‐of‐the‐art performance [6, 22].

### Meniscus localization

The annotation of MRI studies was performed on a total of 1284 images by two orthopaedic surgeons and one radiologist who revised the results. The surgeons have 8 and 10 years of experience, respectively, and the radiologist has 9 years of experience. The agreement between the surgeons and the radiologist was assessed, and the radiologist revised the annotations in 100% of the cases.

**Figure 1:**
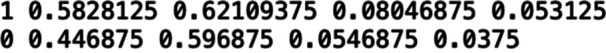
Example of YOLO annotated format showing how the coordinates, height, width and class information are structured for meniscus identification in magnetic resonance imaging.

The annotation process involved labelling the meniscus in the images using Roboflow [4]. Images were annotated in the YOLO format, which provides a systematic approach to object detection. This format includes coordinates for the centre of the object, the height and width of the object and the class of the object. Each meniscus area in the image is identified by the normalized coordinates (*x*, *y*) of its centre, ensuring consistency across different image sizes. The dimensions of the meniscus are specified by its normalized height and width, allowing for accurate detection regardless of the original image resolution. Each annotated area is assigned a class label, indicating that it is a meniscus. Figure 1 illustrates an example of a YOLO annotated format, showing how the coordinates, height, width and class information are structured. This visual representation demonstrates the practical application of the YOLO model in identifying and categorizing meniscal structures within the MRI images, facilitating an accurate and efficient diagnosis of meniscal injuries.

The database has been divided into 450 training, 100 validation and 92 test images for both sagittal and coronal views. Images were integrated into the YOLOv8 model for sagittal and coronal views, respectively, which utilizes a pretrained structure and weights. Images were augmented with horizontal flip, rotation and cropping to provide diversity to training data and prevent overfitting. The model was then trained on our data set for 100 epochs, with an additional 25 epochs dedicated to validation. An epoch refers to one complete pass through the entire training data set. Precision, recall and mAP (mean average precision) values were calculated based on the IoU (intersection over union) metrics.

**Figure 2:**
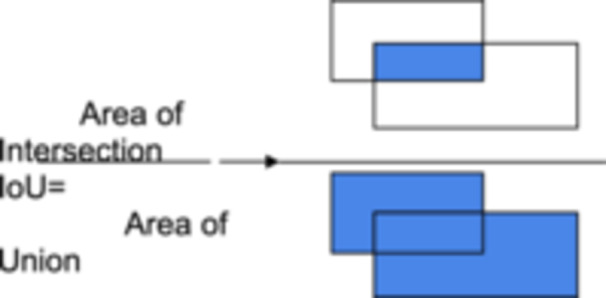
Formula and visual explanation for intersection over union (IoU), which measures the overlap between the predicted bounding box and the ground truth bounding box.

Precision=TP/(TP+FP),

Recall=TP/(TP+FN),

Intersection over union (IoU) measures the overlap between two bounding boxes by dividing the area of their intersection by the area of their union. It is crucial for evaluating object detection models (see Figure 2). Average precision (AP) calculates the area beneath the precision–recall curve, offering a singular metric that summarizes both the precision and recall of a model. The mean average precision (mAP) expands on AP by averaging the AP scores across different object classes, making it valuable for assessing performance in multiclass object detection scenarios comprehensively.

### Meniscus tear detection

For meniscus tear detection, we employed the EfficientNetV2 model and chose the EfficientNet_V2_M_Weights.IMAGENET1K_V1 for pretrained weights due to its balance of compact size and high performance. The model focused on regions of interest (ROIs) within the labelled images, and our data set comprised a total of 1284 images for each plane (642 sagittal and 642 coronal). These images were resized to the input size of 480 × 480 pixels, which is the size the EfficientNetV2‐M model is designed to accept. The resolution was chosen to balance computational efficiency and model performance.

Data processing included resizing the images, normalizing pixel values and applying data augmentation techniques. For optimization, we used the Adam optimizer for gradient descent and the CrossEntropy loss function, incorporating weighted loss to address the class imbalance. Precision, recall and area under the curve (AUC) values were calculated. The AUC is a performance metric that measures the ability of a classification model to distinguish between classes, with values ranging from 0 to 1, where a higher AUC indicates better model performance.

### Computational tools

All training experiments were conducted using Python 3.10.12, PyTorch 2.2.1, Scikit‐learn 1.2.2, NumPy 1.25.2, Roboflow 1.1.28 and Matplotlib 3.7.1. Computations were performed using NVIDIA Tesla V100‐SXM2 GPUs.

## RESULTS

**Figure 3:**
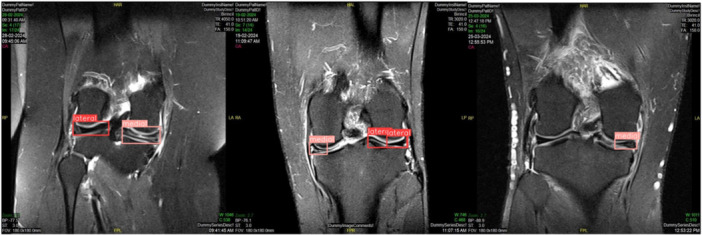
True positive (TP), false positive (FP) and false negative (FN) results in the frontal view of magnetic resonance imaging scans for meniscus tear detection using the YOLOv8 and EfficientNetV2 model.

**Figure 4:**
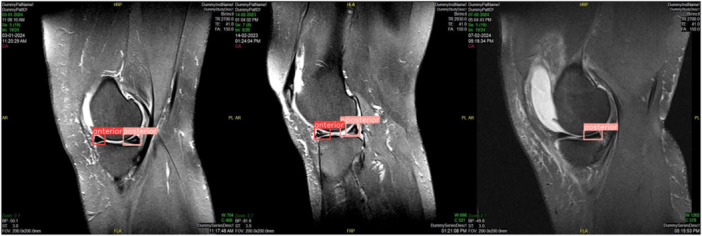
True positive (TP), false positive (FP) and false negative (FN) results in the sagittal view of magnetic resonance imaging scans for meniscus tear detection using the YOLOv8 and EfficientNetV2 model.

The YOLOv8 model achieved high accuracy in meniscus localization, and the EfficientNetV2 model demonstrated strong performance in detecting meniscal tears. Detailed performance metrics are provided in corresponding Table 1 and Figures 3 and 4.

**Table 1:** Study population and their distribution.

Number of patients	642
Female/male ratio (%)	43.1/56.9
Mean age (years)	36.2
Mean weight (kg)	78.3

### Meniscus localization

The precision and recall metrics indicate the model's efficacy in correctly identifying meniscal structures in both sagittal and coronal views. The evaluation of true positives and false positives is presented in Figures 3 and 4.

### Meniscal tear characterization

**Figure 5:**
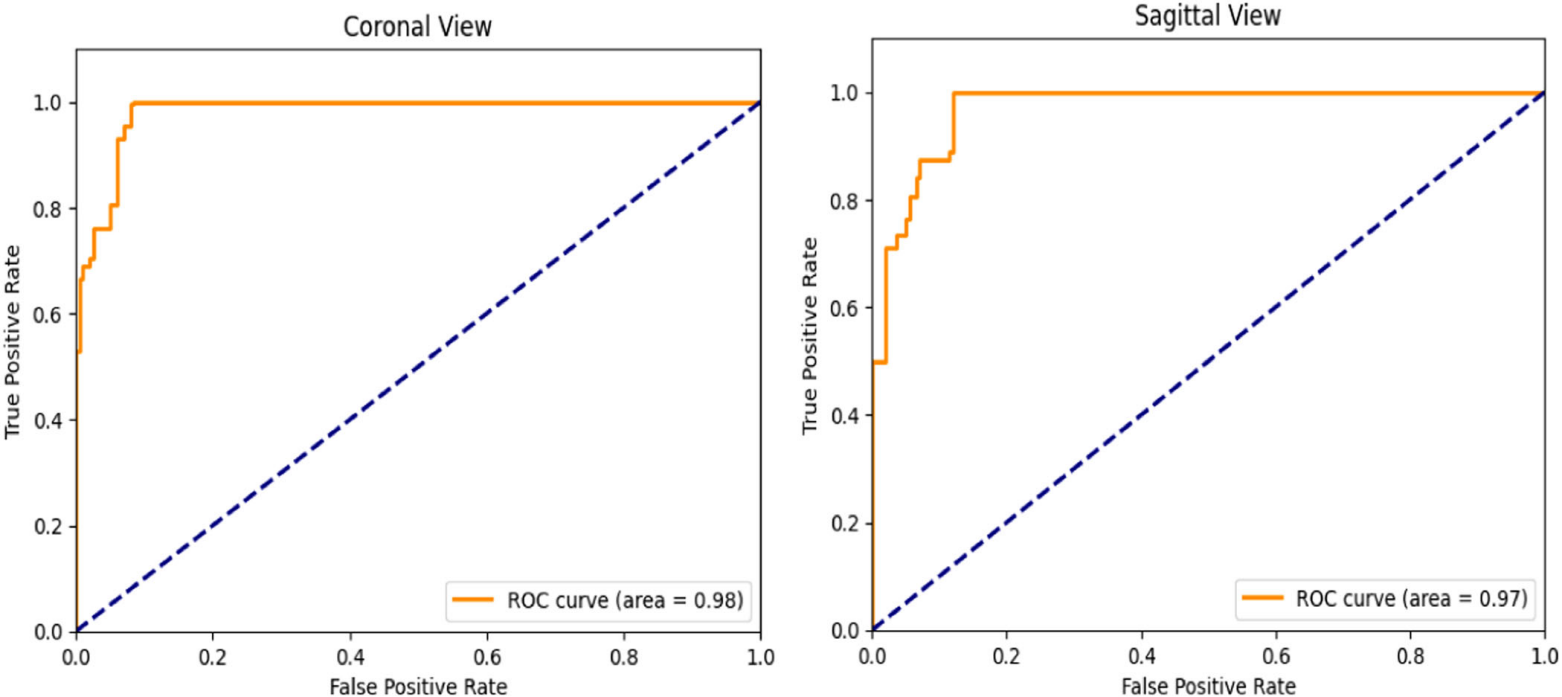
Area under the curve for meniscal tear detection, illustrating the performance of the EfficientNetV2 model in distinguishing between torn and healthy meniscus tissues.

The EfficientNetV2 model has exhibited outstanding classification capabilities in differentiating between torn and healthy meniscus tissues. For sagittal cropped images, the model achieved precision, recall and AUC values of 0.97 each. In the case of coronal cropped images, the model further demonstrated its robustness with a precision of 0.97, a recall of 0.98 and an AUC of 0.98 (Figure 5).

## DISCUSSION

The most important finding of this study was the significant potential of utilizing advanced deep learning models, specifically YOLOv8 and EfficientNetV2, for the detection and localization of meniscal tears in MRI images using a relatively small data set. These findings are particularly noteworthy, given the relatively small data set, highlighting the efficiency and power of state‐of‐the‐art deep learning algorithms. Integrating these cutting‐edge models offers a novel dual‐stage diagnostic approach that outperforms traditional methods, with higher AUC scores and lower false‐positive rates, making it a practical and innovative solution in medical imaging. Our study addresses significant gaps in the literature by providing a feasible tool for healthcare facilities with limited data, thereby improving patient care and operational efficiency. This substantial improvement over existing models and our approach's practical applicability underscores our research's originality and necessity. Unlike previous studies requiring large data sets, our approach demonstrates high accuracy with limited data, making it more feasible for clinical use.

The importance of integrating deep learning technologies into modern clinical practice is evident in recent studies. For example, Oeding et al. provided a practical guide for orthopaedic surgeons, emphasizing how these technologies can significantly improve diagnostic accuracy and operational efficiency [15, 16]. Similarly, Kunze et al. demonstrated the potential of deep learning in analyzing radiographic findings related to knee osteoarthritis, highlighting its relevance in modern orthopaedic diagnostics [12]. Ko et al. outlined three strategies for deep learning with orthopaedic‐specific imaging, reinforcing the practical applications of AI in orthopaedics [11]. Bálint Zsidai et al. discussed the implementation of artificial intelligence in orthopaedic research, providing a technical introduction that complements our findings and underscores the potential of AI in this field [23].

Previous research has explored various AI models for meniscus tear detection, each with different methodologies and results [7, 13, 14]. Furthermore, other studies have demonstrated the broad applicability of deep learning in orthopaedic diagnostics. Borjali et al. showed that deep learning could achieve high diagnostic accuracy in detecting syndesmotic instability on weight‐bearing computed tomography scanning [2]. As demonstrated by Kara and Hardalaç, progressively operating deep learning models, such as their novel ResNet50 model, have been developed to detect meniscus injuries, ACL tears and knee abnormalities in MRI. Their work shows that these models achieve high accuracy, highlighting the potential for deep learning to enhance diagnostic precision and efficiency in clinical settings [8]. Rizk et al. employed two three‐dimensional (3D) convolutional neural network (CNN)‐based models to locate meniscal structures and classify lesions, obtaining AUC scores of 0.93 for medial meniscal tears and 0.84 for lateral meniscal tears [19]. Roblot et al. applied a fast region‐based CNN model to identify the presence and location of meniscus tears, achieving a weighted AUC score of 0.90. However, their training data set was limited to just two specifically chosen T2‐weighted sagittal images [20]. Pedoia et al. implemented a U‐Net‐based region detection model followed by a 3D CNN‐based model for classifying meniscus lesions, achieving an AUC score of 0.89 [17]. Key et al. discovered an alternative method for feature extraction, achieving a rupture classification accuracy of 98.42% with coronal data sets and 100% with sagittal data sets [9]. Chou et al. used Scaled YOLOv4 for localizing the meniscus and EfficientNet‐B7 for detecting tears, achieving AUC scores of 0.948 and 0.963 in the sagittal and coronal views, respectively. The EfficientNet‐B7 model alone yielded AUC scores of 0.984 in the sagittal view and 0.972 in the coronal view [3]. Bien et al. utilized MRNet, a CNN‐based model that analyzes three MRI series (axial, coronal and sagittal), to identify meniscus tears, achieving an AUC score of 0.847 [1]. Deep‐learning‐based approaches have monopolized knee injury detection in MRI studies, achieving prediction accuracies ranging from 72.5% to 100%. These models have the potential to perform at the human level in decision‐making tasks related to MRI‐based diagnosis of knee injuries, demonstrating significant promise in improving the accuracy and cost‐effectiveness of knee injury detection [21]. Our study achieved AUC scores of 0.97 and 0.98 for sagittal and coronal views, respectively, indicating superior performance. This highlights the efficacy of YOLOv8 and EfficientNetV2 in accurately detecting meniscal tears even with a smaller data set, showcasing the advancements in deep learning algorithms.

One limitation of our study is the small data set, which may not fully represent the diversity of meniscal tear presentations. Additionally, using only two slices per patient (one sagittal and one coronal) may not capture the full extent of meniscal injuries, limiting the model's ability to generalize across different types and severities of tears. Future research should include larger and more varied data sets with multiple slices per patient to validate and enhance the model's performance. Moreover, continuous refinement and updating with new data are essential to maintaining clinical relevance and accuracy.

## CONCLUSION

In summary, a deep learning‐based AI system for report generation was developed to identify meniscal injuries. This model could significantly enhance the diagnostic process for meniscal injuries through MR images. By using this AI detection system, clinicians can instantly generate a structured report of meniscus ruptures at the push of a button, facilitating faster image interpretation and saving time. This allows trained medical staff to quickly detect meniscus tears, while less experienced clinicians and other subspecialists can gather diagnostic evidence and reduce the overall physician workload. Future studies will focus on enhancing the AI model's performance through fine‐tuning and the inclusion of additional image data.

## AUTHOR CONTRIBUTIONS

*Conception*: Erdal Güngör and Ahmetcan Cansın. *Design*: Erdal Güngör, Ahmetcan Cansın and Husam Vehbi. *Supervision*: Erdal Güngör and Mehmet Batu Ertan. *Fundings*: Erdal Güngör, Ahmetcan Cansın and Husam Vehbi. *Materials*: Erdal Güngör, Husam Vehbi and Mehmet Batu Ertan. *Data collection and/or processing*: Erdal Güngör and Husam Vehbi. *Analysis and/or interpretation*: Erdal Güngör. *Literature review*: Erdal Güngör. *Writer*: Erdal Güngör and Ahmetcan Cansın. *Critical review*: Erdal Güngör, Ahmetcan Cansın and Mehmet Batu Ertan.

## CONFLICT OF INTERESTS STATEMENT

The authors declare no conflict of interest.

## ETHICS STATEMENT

The study protocol adhered to the principles outlined in the Declaration of Helsinki and received approval from the local ethical committee of our hospital (Decision no: 271, Date: March 14, 2024).

## Data Availability

The data sets used and/or analysed during the current study are available from the corresponding author upon reasonable request.
